# Professor Austin Flint’s Fever Reports

**Published:** 1851-11

**Authors:** 


PROFESSOR AUSTIN FLINT'S FEVER REPORTS.

The able and industrious editor of the *Buffalo Medical Journal* is publishing a series of statistical investigations, preparatory to "the *diagnosis, identity of Typhus and Typhoid, and the treatment*," which is eminently worthy of the attention of medical readers. Professor Flint prays the patience of his readers towards his series, but we think the patients of his medical brethren will gain considerably if their Doctors read Dr. Flint's papers with care. We intend to wait until the series is complete, before we criticise. We may then break a lance with Professor Flint on several points towards which his investigations are evidently tending. B.

